# Microbiome networks and change-point analysis reveal key community changes associated with cystic fibrosis pulmonary exacerbations

**DOI:** 10.1038/s41522-018-0077-y

**Published:** 2019-01-21

**Authors:** Mehdi Layeghifard, Hannah Li, Pauline W. Wang, Sylva L. Donaldson, Bryan Coburn, Shawn T. Clark, Julio Diaz Caballero, Yu Zhang, D. Elizabeth Tullis, Yvonne C. W. Yau, Valerie Waters, David M. Hwang, David S. Guttman

**Affiliations:** 1 Department of Cell and Systems Biology, University of Toronto, Toronto, ON Canada; 2 Department of Biochemistry, University of Toronto, Toronto, ON Canada; 3 Centre for the Analysis of Genome Evolution and Function, University of Toronto, Toronto, ON Canada; 4 Department of Laboratory Medicine and Pathobiology, University of Toronto, Toronto, ON Canada; 5 Latner Thoracic Surgery Laboratories, University Health Network, University of Toronto, Toronto, ON Canada; 6 Adult Cystic Fibrosis Clinic, St. Michael’s Hospital, Toronto, ON Canada; 7 Department of Pediatric Laboratory Medicine, Microbiology, The Hospital for Sick Children, Toronto, ON Canada; 8 Department of Laboratory Medicine and Pathobiology, University of Toronto, Toronto, ON Canada; 9 Division of Infectious Diseases, Department of Pediatrics, Hospital for Sick Children, University of Toronto, Toronto, ON Canada; 10 Department of Pathology, University Health Network, University of Toronto, Toronto, ON Canada

## Abstract

Over 90% of cystic fibrosis (CF) patients die due to chronic lung infections leading to respiratory failure. The decline in CF lung function is greatly accelerated by intermittent and progressively severe acute pulmonary exacerbations (PEs). Despite their clinical impact, surprisingly few microbiological signals associated with PEs have been identified. Here we introduce an unsupervised, systems-oriented approach to identify key members of the microbiota. We used two CF sputum microbiome data sets that were longitudinally collected through periods spanning baseline health and PEs. Key taxa were defined based on three strategies: overall relative abundance, prevalence, and co-occurrence network interconnectedness. We measured the association between changes in the abundance of the key taxa and changes in patient clinical status over time via change-point detection, and found that taxa with the highest level of network interconnectedness tracked changes in patient health significantly better than taxa with the highest abundance or prevalence. We also cross-sectionally stratified all samples into the clinical states and identified key taxa associated with each state. We found that network interconnectedness most strongly delineated the taxa among clinical states, and that anaerobic bacteria were over-represented during PEs. Many of these anaerobes are oropharyngeal bacteria that have been previously isolated from the respiratory tract, and/or have been studied for their role in CF. The observed shift in community structure, and the association of anaerobic taxa and PEs lends further support to the growing consensus that anoxic conditions and the subsequent growth of anaerobic microbes are important predictors of PEs.

## Cystic Fibrosis: Oxygen, microbes and exacerbation

Episodes of significant worsening of cystic fibrosis symptoms, known as pulmonary exacerbations (PEs), are associated with oxygen-deficient (anoxic) conditions and increased activity of ‘anaerobic’ bacteria, which thrive in the absence of oxygen. Researchers in Canada, led by David Guttman at the University of Toronto, compared genetic data on microbial populations in sputum samples collected during PEs and at times of better health. The study revealed a strong correlation between the activity and interactions among anaerobic bacteria and the onset of PEs. Investigating the significance of these changes in the lung environment and its microbial populations may help design treatment strategies to reduce the frequency of PEs and their potentially fatal consequences. The authors suggest that antibiotics that specifically target anaerobic bacteria may prove beneficial, as may hyperbaric oxygen therapy, which oxygenates the lung tissue.

## Introduction

Cystic fibrosis (CF) is a systemic, genetic disease accompanied by chronic airway infections largely caused by defective mucociliary clearance.^1^ The most significant declines in CF patient health occur during intermittent, acute, pulmonary exacerbations (PEs), which are physician-defined clinical events that typically require additional antibiotic treatment.^2^^–5^ Given the significant impact of PEs on patient health, a major motivating factor driving CF microbiome research has been to better understand the interrelationship between the CF lung microbiome and PEs, with the hope of identifying novel therapeutic strategies or predictive biomarkers that will enable physicians to reduce the frequency and/or severity of PEs. Despite this large body of work, few studies have discovered microbiological signals associated with PEs,^6–14^ and the underlying causes of PEs are still largely unknown.^3,15^

One of the most intriguing associations between the CF lung microbiome and PEs identified to date comes from Quinn and colleagues^13^ who used network analysis on both taxonomic and inferred metabolic data obtained from 126 CF sputum samples to identify distinct microbial clusters. This cross-sectional study found that taxa clustered into three distinct groups. The first network cluster was dominated by anaerobic microbes and was negatively associated with two other network clusters, which were dominated by classic CF pathogens such as *Pseudomonas* and *Staphylococcus*. The finding of distinct ecological clusters or communities supports the recently proposed Climax - Attack Model of CF lung microbial diversity,^13,16–21^ which postulates the existence of climax and attack microbiomes that are functionally distinct, but not mutually exclusive. Climax microbiomes describe persistent communities that dominate during periods of clinical stability, and which are enriched for antibiotic resistant microbes expressing genes that promote chronic colonization. Attack microbiomes, on the other hand, are more transient communities associated with early lung colonization and PEs.^13^

The interplay among and between microbes and the CF lung provides insight into disease progression and ecological dynamics. For example, the initial colonization of the CF lung by the classic CF pathogens (e.g., *Pseudomonas aeruginosa, Burkholderia cepacia* complex, and *Staphylococcus aureus*) is believed to elicit an immune response resulting in inflammation and mucus hyper-concentration. This response creates a remodeled lung environment conducive to the establishment of chronic infections by these pathogens.^17,21^ Subsequently, the members of the chronic infection community have been shown to form aggregates in CF sputum *in vitro*,^22^ and it is speculated that this aggregation *in vivo* results in biofilms that impair mucociliary clearance and effectively plug regions of the lung.^21^ Obstructed regions in the lung experience decreased oxygen tension, which favors the fermentative growth of anaerobes and the production of fermentation metabolites such as acetaldehyde and 2,3-butanedione.^18,19,23^ Acidic fermentation products lower lung pH, which further damages the lung tissue creating the potential for a feedback loop. Ultimately, this damage is believed to increase the likelihood of PEs^24–26^; thereby, creating a direct link between environmental modeling caused by the chronic infection pathogens (e.g., biofilms), the subsequent growth of anaerobic microbes, and PEs.

Here we introduce an unsupervised network approach to identify key members of the CF lung microbiome associated with specific clinical states with the goal of better understanding how microbial communities change during the course of disease development. Key taxa are those that have an elevated influence on their environment regardless of their relative abundance.^27^ Our approach has the following two main objectives: (1) identify key microbial taxa using taxa co-occurrence (i.e. ecological) networks and hub detection, and (2) assess associations between changes in the abundance of key taxa and changing patterns of patient health over time using change-point detection. The former is important due to its focus on highly interconnected taxa, rather than on simply those taxa that are most abundant or prevalent. The latter is important since change-point detection provides a statistical framework for assessing correlated changes in the abundance of key taxa and patient clinical state over time. We examined CF lung microbiome dynamics on a longitudinal collection of 266 expectorated sputum samples obtained from 18 CF patients and show that changes in the abundances of highly interconnected (e.g., hub) taxa track clinical changes more closely than the taxa with either the highest relative abundance or prevalence. We also show that anaerobic taxa are strongly associated with PEs, providing additional support to the growing consensus that the development of anoxic conditions in the CF lung and the subsequent role of anaerobic microbes is an important factor leading to PEs.

## Results

### Study collections

We used two independent CF lung microbiome data sets in this study. The first (i.e., discovery) data set, was composed of 266 expectorated sputum samples obtained from 18 CF patients (range 8–60 samples / patient, mean = 14.1 ± 11.92 s.d., median = 11) being treated at St. Michael’s Hospital and the Hospital for Sick Children in Toronto, Canada. They were interrogated at the V5 to V7 hypervariable regions of the 16S rRNA gene and analyzed following the protocol described by Maughan et al.^28^ Patients had an average of 0.76 ± 1.044 s.d. (median = 1.04) PEs over the course of sampling, with sampling interval time averaging 654 ± 257 s.d. (median = 640) days. Each sample was categorized into one of the following three categories (i.e., clinical states) based on the physician’s diagnosis at the time of the patient's clinic visit: (a) baseline, (b) unwell, and (c) PE. We also used an alternative clinical classification scheme proposed by Zhao et al.^10^ known as the BETR system to confirm that our conclusions were not the result of bias due to the classification method.

We validated our finding using an entirely independent microbiome data set obtained from Carmody et al.^11^ This validation data set was composed of daily sputum samples collected from four CF patients who experienced PE during the course of sampling, and were collected by John LiPuma and colleagues at the University of Michigan (Ann Arbor, MI). The microbiome data were generated using the V3-V5 hypervariable region of the 16S rRNA locus following different amplification, sequencing, and analysis protocols. Since these data are as independent as possible, they provide the most rigorous assessment of the power and robustness of our analytical approach. Validation data sets also ensure that biases in any one data set do not unduly skew the resulting conclusions.

### Identifying key taxa associated with clinical changes via longitudinal analysis

**Fig. 1:**
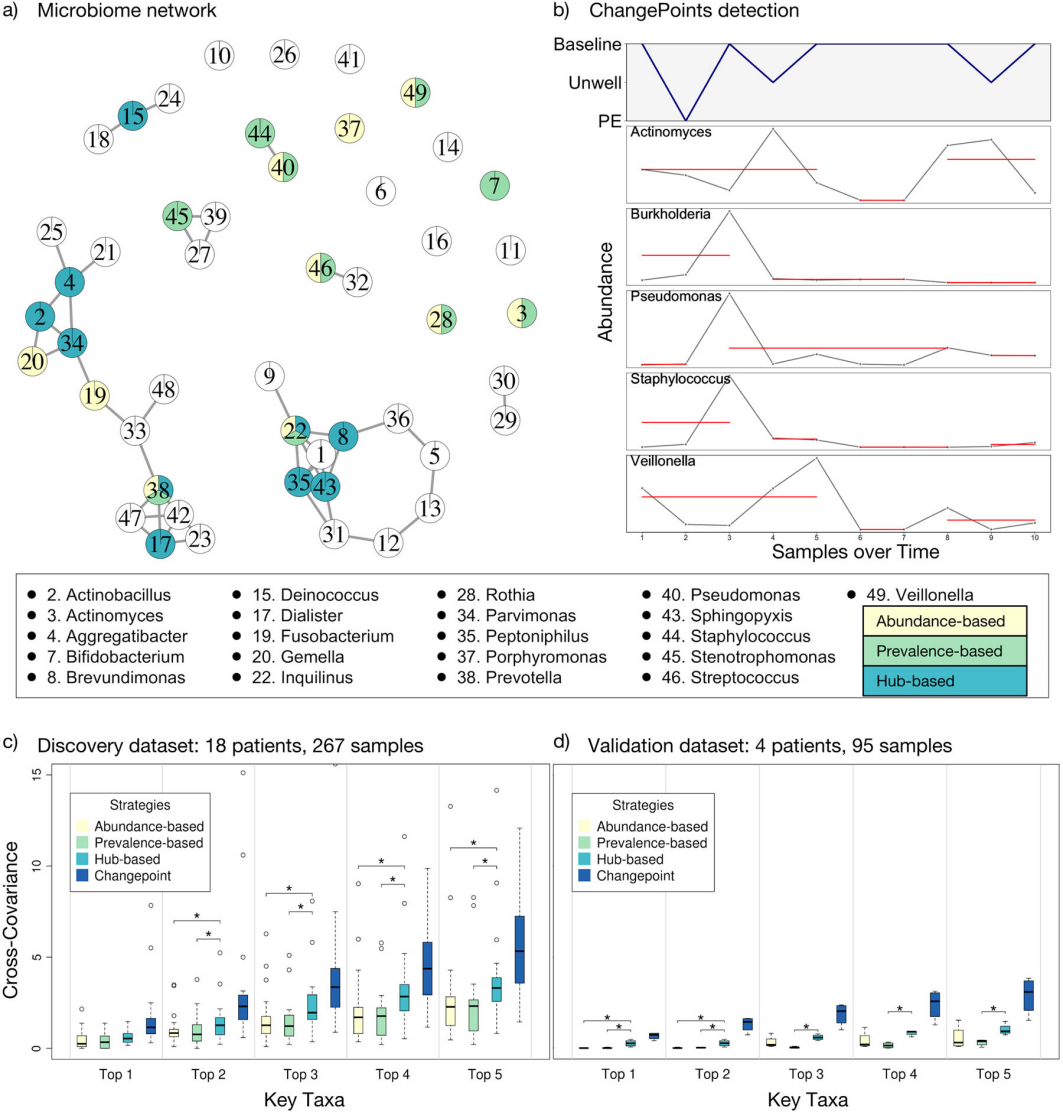
Association between key taxa and clinical states of CF patients. Key taxa were identified using abundance-based, prevalence-based, and hub-based strategies as described in the text. **a** A representative network of a single patient’s microbiome with hubs representing taxa at the genus level and edges representing co-occurrence. The top ten key taxa detected by the three strategies are highlighted in the network using colored pie charts with yellow, green, and blue identifying taxa as top ten via abundance-based, prevalence-based, or hub-based methods, respectively. The names of the top 10 key taxa identified by any of the three strategy are shown in the key on the bottom. **b** Changes in the relative abundance of well-known taxa over time compared to changes in the clinical state of a single patient. The solid red lines represent the samples collected over time that do not significantly change in their abundances. Breaks in the red lines indicate changes in abundance identified by the change-point detection algorithm. **c** Cumulative cross-covariance measures of association between key taxa and clinical states of all CF patients in the discovery data set (longitudinal microbiome data from 18 CF patients). The boxes are interquartile range boxes representing the middle 50% of the data values. The line in the middle of each box represents the median (i.e. middle quartile). The whiskers extending from either side of each box represent the ranges for the top and bottom 25% of the data, excluding outliers which are shown as hollow points. The y-axis represents the cumulative sum of cross-covariances for the identified key taxa. The higher this measure, the stronger is the association between changes in the abundances of the top key taxa and changes in the clinical states. The *x* axis represents top one to top five ranked key taxa found by the four strategies, such that the ‘Top *n*’ category (where, *1* *<* *=* *n* *>* *=* *5*) shows the sum of the cross-covariance measures of the *n* most important taxa. Thus, we expect an increase in the cumulative cross-covariance measures with increasing number of key taxa, *n*. We also identified a change-point standard for each patient consisted of the 10 taxa whose changes in abundance showed the strongest association to changes in clinical state. The Mann-Whitney-Wilcoxon test was used for statistical hypothesis testing (*p* < 0.05; The asterisks represent the significant differences between the strategies). **d** Validation data set from Carmody et al.^11^ including longitudinal data from four patients is presented in the same manner as described in **c**

We employed the following three strategies to identify key taxa: (a) an **abundance-based** strategy that identifies key taxa as those with the highest overall relative abundance (i.e., abundance-based, AB); (b) a **prevalence-based** strategy that identifies key taxa as those with the highest prevalence across all samples (i.e., prevalence-based, PB); and (c) a **hub-based** strategy that identifies key taxa as those that are most central to the microbiome networks (i.e., highest degree of interconnectedness, hub-based, HB). For the last strategy, we constructed a microbiome co-occurrence network using the OTU data from each patient with each OTU resolved to the genus level. The co-occurrence networks were constructed via FDRnetwork method from qgraph R package,^29^ and the top-ranked network hub taxa were identified by PageRank algorithm.^30^ For each strategy, we recorded both the top five and top ten taxa for further analysis. Figure 1a shows a microbiome network for a single representative patient with the key taxa identified by each of the three strategies. The relative abundances and ranking of the top ten taxa identified by the three strategies are presented in Tables 1–3, while the full data are available in the Supplementary Table 2.

**Table 1:** Top ten genera by network interconnectedness (Hubs) stratified by clinical state

	Rank	Genus	Mean (%)	Median (%)	Stdev	Prev. (%)	RA rank	Prev. rank	Metab.^a^
Baseline	1	*Johnsonella*	0.098	0.016	0.206	72.5	29	18	−
	2	*Veillonella*	3.455	1.460	4.994	95.0	6	5	−
	3	*Propionibacterium*	0.023	0.005	0.052	77.5	43	16	−
	4	*Prevotella*	12.537	6.257	15.089	100.0	2	1	−
	5	*Pseudomonas*	41.289	46.880	36.709	92.5	1	7	+
	6	*Bifidobacterium*	0.733	0.020	2.750	77.5	15	15	−
	7	*Derxia*	0.024	0.000	0.153	12.5	42	55	+
	8	*Mogibacterium*	0.111	0.002	0.333	55.0	28	32	−
	9	*Aeromonas*	0.000	0.000	0.001	6.3	94	70	+
	10	*Streptococcus*	9.705	5.769	11.204	98.8	3	3	+
Unwell	1	*Mycoplasma*	0.007	0.000	0.068	5.3	56	80	−
	2	*Halomonas*	0.001	0.000	0.003	18.1	94	51	+
	3	*Enterococcus*	1.715	0.006	5.806	57.9	11	25	+/−
	4	*Solobacterium*	0.499	0.035	1.812	72.5	16	10	−
	5	*Helicobacter*	0.005	0.000	0.033	8.8	61	62	+
	6	*Pseudomonas*	30.790	13.553	34.454	84.2	1	7	+
	7	*Bifidobacterium*	1.575	0.010	5.768	68.4	12	13	−
	8	*Capnocytophaga*	0.139	0.004	0.392	57.3	29	28	+/−
	9	*Stenotrophomonas*	9.389	0.096	18.196	87.7	4	6	+
	10	*Porphyromonas*	1.060	0.002	5.884	57.9	14	26	−
PE	1	*Actinomyces*	2.311	0.517	3.661	100.0	9	6	−
	2	*Bergeyella*	0.010	0.000	0.018	31.3	55	50	+
	3	*Leptotrichia*	0.039	0.004	0.072	75.0	37	25	−
	4	*Selenomonas*	0.015	0.000	0.027	31.3	49	49	−
	5	*Treponema*	0.004	0.000	0.011	18.8	63	61	+/−
	6	*Dialister*	0.046	0.002	0.127	50.0	36	39	−
	7	*Propionibacterium*	0.014	0.001	0.020	62.5	50	30	−
	8	*Johnsonella*	0.133	0.041	0.303	81.3	28	19	−
	9	*Prevotella*	9.927	7.086	12.885	100.0	3	2	−
	10	*Solobacterium*	0.077	0.020	0.137	87.5	31	15	−

*Prev* Prevalence, *RA* relative abdundance

^a^Metab. Metabolism: + aerobic, − anaerobic +/− facultative anaerobic

**Table 2:** Top ten genera by relative abundance (ra) stratified by clinical state

	Rank	Genus	Mean (%)	Median (%)	Stdev	Prev. (%)	Prev. rank	Hub rank	Metab.^a^
Baseline	1	*Pseudomonas*	41.289	46.880	36.709	92.5	7	5	+
	2	*Prevotella*	12.537	6.257	15.089	100.0	1	4	−
	3	*Streptococcus*	9.705	5.769	11.204	98.8	3	10	+
	4	*Rothia*	6.383	2.002	13.623	100.0	2	54	−
	5	*Stenotrophomonas*	6.289	0.023	20.681	92.5	8	23	+
	6	*Veillonella*	3.455	1.460	4.994	95.0	5	2	−
	7	*Inquilinus*	2.990	0.000	11.405	18.8	52	78	+/−
	8	*Actinomyces*	2.419	0.666	4.089	98.8	4	19	−
	9	*Haemophilus*	2.364	0.006	10.865	70.0	19	20	+/−
	10	*Fusobacterium*	1.542	0.101	3.562	82.5	12	22	−
Unwell	1	*Pseudomonas*	30.790	13.553	34.454	84.2	7	6	+
	2	*Prevotella*	15.143	6.885	19.744	96.5	3	16	−
	3	*Streptococcus*	12.074	5.016	14.122	98.8	1	25	+
	4	*Stenotrophomonas*	9.389	0.096	18.196	87.7	6	9	+
	5	*Rothia*	5.884	1.776	12.203	98.2	2	14	−
	6	*Veillonella*	4.898	0.886	8.430	93.6	4	11	−
	7	*Burkholderia*	4.136	0.009	18.832	66.1	16	128	+
	8	*Staphylococcus*	2.631	0.015	9.910	81.3	8	99	+/−
	9	*Actinomyces*	2.138	0.217	4.041	93.0	5	19	−
	10	*Inquilinus*	1.854	0.000	9.889	19.3	48	33	+/−
PE	1	*Pseudomonas*	36.645	33.068	36.134	93.8	9	30	+
	2	*Streptococcus*	14.127	9.869	13.198	100.0	1	22	+
	3	*Prevotella*	9.927	7.086	12.885	100.0	2	9	−
	4	*Rothia*	6.466	0.480	15.744	100.0	3	35	−
	5	*Fusobacterium*	4.849	0.183	11.018	75.0	20	64	−
	6	*Haemophilus*	4.755	0.023	17.451	100.0	4	52	+/−
	7	*Inquilinus*	3.512	0.000	8.686	31.3	47	67	+/−
	8	*Veillonella*	2.894	1.069	3.511	100.0	5	11	−
	9	*Actinomyces*	2.311	0.517	3.661	100.0	6	1	−
	10	*Stenotrophomonas*	2.275	0.092	5.406	93.8	10	29	+

^1^ Prev. prevalence

^a^Metab. = Metabolism: + aerobic; − anaerobic; +/− facultative anaerobic

**Table 3:** Top ten genera by prevalence stratified by clinical state

	Rank	Genus	Mean (%)	Median (%)	Stdev	Prev. (%)	RA rank	Hub rank	Metab.^a^
Baseline	1	*Prevotella*	12.537	6.257	15.089	100.0	2	4	−
	2	*Rothia*	6.383	2.002	13.623	100.0	4	54	−
	3	*Streptococcus*	9.705	5.769	11.204	98.8	3	10	+
	4	*Actinomyces*	2.419	0.666	4.089	98.8	8	19	−
	5	*Veillonella*	3.455	1.460	4.994	95.0	6	2	−
	6	*Gemella*	1.528	0.393	2.769	95.0	11	11	+/−
	7	*Pseudomonas*	41.289	46.880	36.709	92.5	1	5	+
	8	*Stenotrophomonas*	6.289	0.023	20.681	92.5	5	23	+
	9	*Staphylococcus*	1.076	0.011	4.598	90.0	14	41	+/−
	10	*Solobacterium*	0.485	0.041	1.034	85.0	18	25	−
Unwell	1	*Streptococcus*	12.074	5.016	14.122	98.8	3	25	+
	2	*Rothia*	5.884	1.776	12.203	98.2	5	14	−
	3	*Prevotella*	15.143	6.885	19.744	96.5	2	16	−
	4	*Veillonella*	4.898	0.886	8.430	93.6	6	11	−
	5	*Actinomyces*	2.138	0.217	4.041	93.0	9	19	−
	6	*Stenotrophomonas*	9.389	0.096	18.196	87.7	4	9	+
	7	*Pseudomonas*	30.790	13.553	34.454	84.2	1	6	+
	8	*Staphylococcus*	2.631	0.015	9.910	81.3	8	99	+/−
	9	*Gemella*	1.306	0.020	3.857	81.3	13	12	+/−
	10	*Solobacterium*	0.499	0.035	1.812	72.5	16	4	−
PE	1	*Streptococcus*	14.127	9.869	13.198	100.0	2	22	+
	2	*Prevotella*	9.927	7.086	12.885	100.0	3	9	−
	3	*Rothia*	6.466	0.480	15.744	100.0	4	35	−
	4	*Haemophilus*	4.755	0.023	17.451	100.0	6	52	+/−
	5	*Veillonella*	2.894	1.069	3.511	100.0	8	11	−
	6	*Actinomyces*	2.311	0.517	3.661	100.0	9	1	−
	7	*Staphylococcus*	0.474	0.099	0.984	100.0	17	36	+/−
	8	*Neisseria*	0.228	0.014	0.488	100.0	26	23	+
	9	*Pseudomonas*	36.645	33.068	36.134	93.8	1	30	+
	10	*Stenotrophomonas*	2.275	0.092	5.406	93.8	10	29	+

*Prev.* prevalence, *RA* relative abundance

^a^Metab. = Metabolism: + aerobic, − anaerobic, +/− facultative anaerobic

We used the discovery data set to examine all longitudinal samples collected from each patient and identified abundance change-points, which are time-points in a longitudinal series where the abundance of the key taxa changed significantly. This analysis was done for both the top five and top ten key taxa identified by the three strategies (i.e., AB, PB, & HB). We then used a maximum likelihood-based change-point detection algorithm called pruned exact linear time (PELT)^31^ on each of the key taxa to determine when there were significant changes in their abundance over time. We also applied change-point analysis to each patient’s clinical data to identify significant changes in clinical state over time. Figure 1b shows a plot of the clinical state of one representative patient paired with the changes in the abundance of some well-known CF lung microbes over time.

Finally, we estimated a cross-covariance measure of association between changes in each patient’s clinical state and changes in the abundance of each key taxon over time. Cross-covariance is a statistical measure of association in the relative timing of two temporal signals. We also identified the taxa whose changes in abundance showed the highest degree of association to changes in clinical state for each patient, which we called the change-point-based standard (CB; dark blue bars in Fig. 1c–d). It needs to be noted that the three major strategies used to identify key taxa (i.e. AB, PB, HB; yellow, green and light blue bars, respectively in Fig. 1) require no prior knowledge of the patient’s clinical state, while the change-point-based standard does require prior clinical information, and is therefore not appropriate for predictive or diagnostic methods. Nevertheless, the change-point-based standard does provide a reference for assessing the performance of the other three strategies.

Figure 1c shows the cumulative cross-covariance measures estimated for all 18 CF patients from the discovery data set. A non-parametric Mann-Whitney-Wilcoxon test showed that while no method reached the theoretical maximum association found with the change-point-based approach, the hub-based approach tracked clinical changes significantly better than either the abundance- or prevalence-based strategies.

We validated our longitudinal analysis using our independent validation data set provided by Carmody and colleagues.^11^ Figure 1d shows similar cross-covariance measurements for our validation data set of four CF patients.^11^ Similar to the discovery data set, the hub-based approach significantly outperformed the other two strategies. We also validated our approach using an alternative clinical classification scheme proposed by Zhao et al.^10^ known as the BETR system and obtain similar results with the hub-based strategy for selecting key taxa significantly out-performing the other strategies (Supplementary Figure 1).

We used regression analysis in order to understand the association between the discovery and validation data sets. For this analysis, we performed a regression between cross-covariance measurements of key taxa from the two data sets for each strategy separately, as well as for all strategies combined. The coefficient of determination for the regression of the discovery and validation data sets using the top 5 key taxa was 0.89 for the hub-based approach, compared to 0.59 and 0.05 for the abundance-based and prevalence-based approaches, respectively (Supplementary Figure 2). Similar results were obtained when analyzing the top 10 key taxa (Supplementary Figure 3). These results indicate that the hub-based approach identifies a much more consistent group of key taxa across independent and heterogeneous data sets. We interpret this as indicating that putative ecological interactions (i.e. co-occurrence) of different taxa in the microbiome are more stable than the abundance or prevalence of any specific taxa.

### Heterogeneity of key taxa among selection strategy

**Fig. 2:**
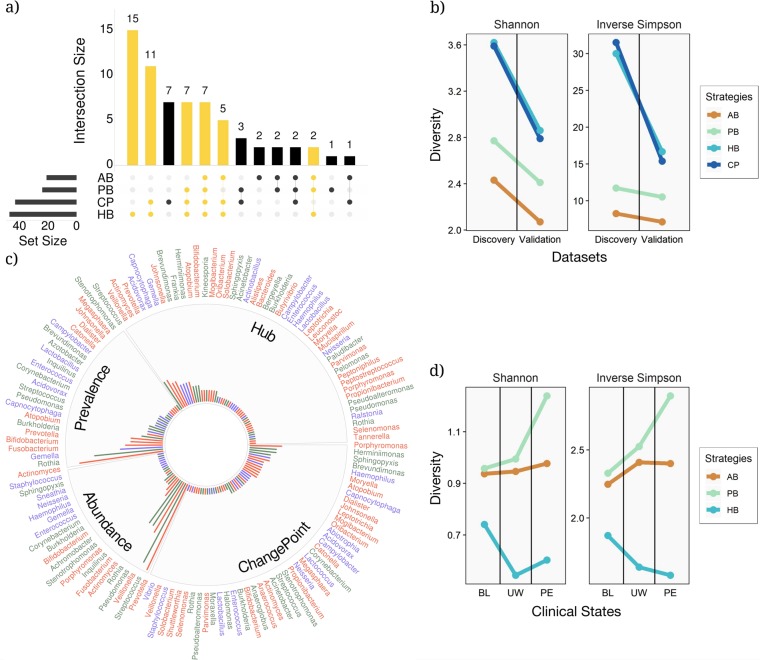
The relationships between the different key taxa selection strategies. **a** An UpSetR visualization of interactions between sets of key taxa identified by the four different key taxa selection strategies within the discovery data set. The grid along the bottom is used to identify interaction sets (analogous to a Venn diagram). A heavily colored yellow or black dot in the grid indicates that the key taxa detection strategy showed on the left has contributed to the interaction set showed on the top. The number of key taxa identified by each strategy and the size of each interaction set are represented, respectively, by horizontal bars on the left and vertical bars on the top. The interaction sets to which the HB strategy has contributed are shown in yellow (AB: abundance-based, PB: prevalence-based, HB: hub-based, and CP: change-point standard). **b** Analysis of diversity for the three key taxa identification strategies using Shannon and inverse Simpson diversity for the discovery and validations data sets. **c** All the key taxa identified in the discovery data set stratified by the key taxa selection strategy. Taxa are color-coded based on their mode of metabolism (green, blue, and red for aerobic, facultative, and anaerobic microbes, respectively). The bar radiation from the center of the chart indicates the relative frequency of each key taxon among all the patients in the data set. **d** Shannon and inverse Simpson diversity plots showing the amount of diversity associated with each of the three clinical states: baseline (BL), unwell (UW), and pulmonary exacerbation (PE)

We examined the relationships among the sets of key taxa identified by each of the three selection strategies for both the discovery and validation data sets to determine which strategy provided results most similar to the change-point standard (i.e. the theoretical best outcome). We examined all key taxa identified by the three strategies and compared these to what was obtained from the change-point standard, plotting via an interactive set visualization approach implemented in UpSetR^32,33^ (Fig. 2a). The hub-based approach identified many more taxa and shared many more of those taxa with the change-point standard than either of the other two selection strategies. Figure 2a shows that the largest shared set is between the hub-based approach and change-point-based standard, with the change-point standard sharing a total of 69.8% of taxa with the hub-based strategy, and only 44.2% and 34.9% of taxa with the prevalence-based and abundance-based strategies, respectively. These results are also supported in the validation data set (Supplementary Figure 4).

We quantified the amount of diversity recovered by the different strategies using the Shannon and Inverse Simpson diversity metrics (Fig. 2b) and found that the hub-based strategy captures substantially more diversity than the other strategies, and is essentially identical to the amount of diversity captured by the theoretical maximum change-point standard. Finally, we found that the hub-based approach detects a more diverse group of taxa among divergent sets of patients when taxa are stratified based on the mode of metabolism (i.e., aerobic, facultative, and anaerobic represented by green, blue and red colors in Fig. 2c).

### Identifying key taxa associated with clinical states via cross-sectional analysis

**Fig. 3:**
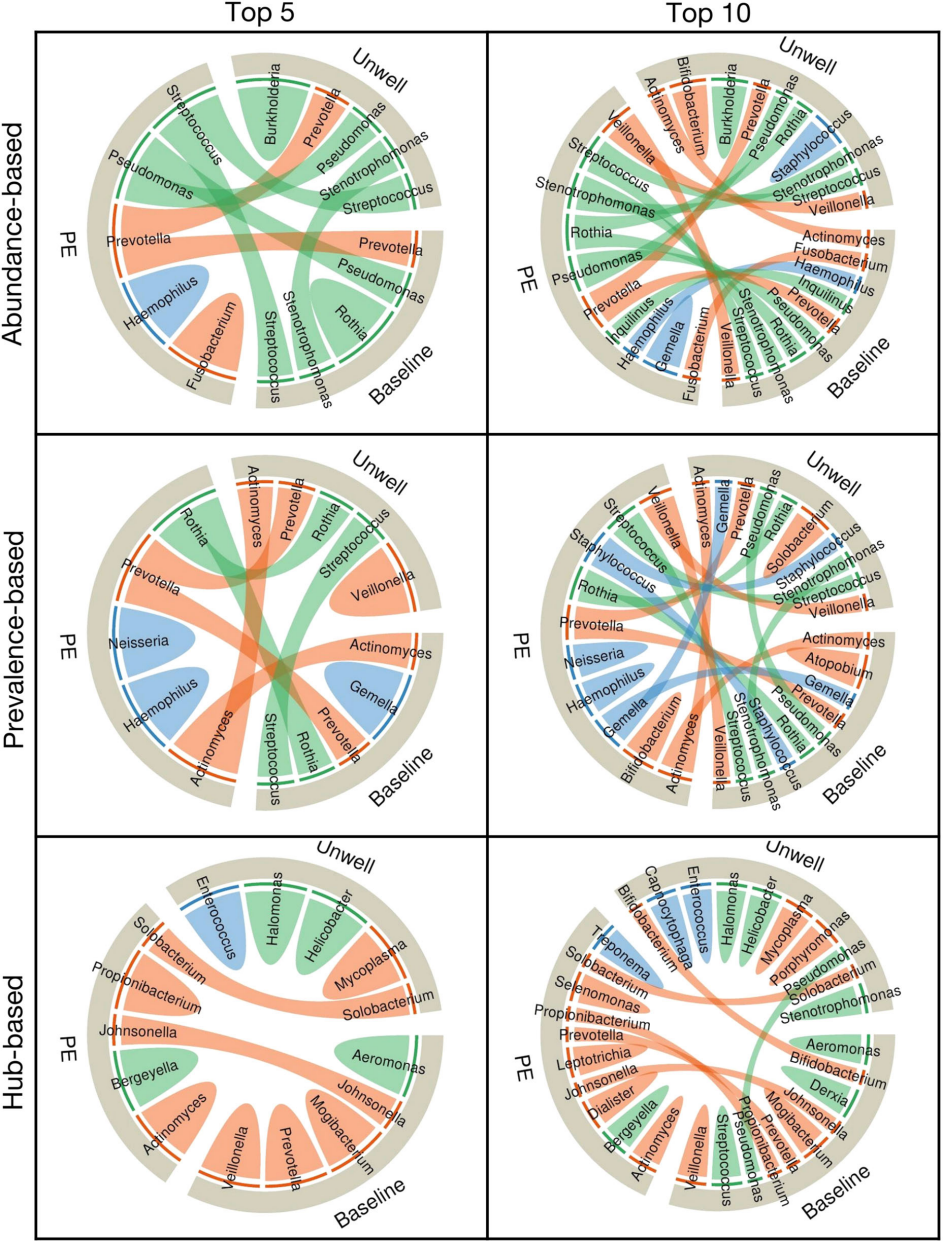
Associations between key taxa of CF lung microbiome and different clinical states in the cross-sectional analysis for both the top five and top ten key taxa. The chord diagrams show the key taxa identified by the three different strategies (e.g., abundance-, prevalence-, or hub-based). The outer ribbon identifies the respective clinical states and encompasses the set of key taxa identified to be associated with each state. Taxa that are associated with more than one state are connected by chords in the inner circle. Taxa and their respective chords are colored based on their primary mode of metabolism, with orange, blue, and green representing anaerobic, facultative anaerobic, and aerobic taxa, respectively. Fewer interactions (i.e. chords) between clinical states demonstrate the higher level of stratification of key taxa based on patients’ health, i.e., the power of each strategy to delineate key taxa based on patients’ clinical states

Next, we performed a cross-sectional analysis of our patient samples to identify key members of the microbiome associated with each clinical state. We assembled the microbiome data from all samples collected at baseline, unwell, and PE states, and determined the top five or ten key taxa for each clinical state based on relative abundance-, prevalence-, and hub-based strategies (Fig. 3). Supplementary Figure 5 shows a network diagram of the data stratified by clinical state as well as the network location of the top ten taxa identified by each strategy. Summary data are presented as chord diagrams in Fig. 3, with each chord diagram divided into three sections representing the three clinical states. Taxa identified as key in two or more clinical states are indicated by arcs (chords) connecting the different clinical states. This analysis reveals that the hub-based strategy more cleanly delineates taxa among the clinical states than the other two strategies, with 84.6% of top five key taxa found in only one clinical category, as compared to 50% of top five key taxa delineated among clinical states for either abundance- or prevalence-based strategies. This can be visualized by the relatively small number of arcs connecting the different clinical states for the hub-based strategy compared to the other strategies in Fig. 3. The hub-based strategy clustered none of the key taxa in all three clinical categories compared to 25% and 37.5% of top five key taxa for abundance- or prevalence-based strategies, respectively. We also performed the analysis on the top ten key taxa using all the three strategies (second column in Fig. 3) and found similar results; the hub-based strategy delineated 75% of the top ten key taxa into a single clinical category, while only 28.5% and 35.7% of abundance- and prevalence-based key taxa were found in one clinical category (Tables 1–3). Again, no hub-based key taxa were identified in all the three clinical categories compared to 50% and 42.8% of the top ten key taxa found by abundance- and prevalence-based strategies. The success of the hub-based strategy highlights the importance of using ecologically-based methods (e.g., taxa co-occurrence networks) to identify key species, and may help explain why prior analyses found few taxa specifically associated with PEs.

We also considered the mode of metabolism of the key taxa and found that anaerobic taxa are over-represented in PEs. Anaerobic and facultative anaerobic taxa are shown in orange and blue, respectively, in the chord diagrams of Fig. 3. The hub-based strategy found that 90% of the top ten key taxa in the PE clinical state are either anaerobes or facultative anaerobes, while representing only 60% of the top ten taxa in the baseline and unwell states. In comparison, 50% of the top ten key taxa are anaerobes or facultative anaerobes in the PE clinical state for the abundance-based strategy, while 80% are anaerobes or facultative anaerobes for the prevalence-based strategy.

We repeated the analysis using the validation data set and an alternative clinical categorization scheme to assess the robustness of our conclusions. Supplementary Figure 6 shows cord diagrams for the validation data set. In this case, the authors only stratified their samples into two categories: baseline, and PE. Supplementary Figure 7 shows an analysis of the discovery data set when the samples are stratified into the four BETR (baseline, PE, treatment, recovery) categories. Both analyses provide similar results, supporting the finding of an over-abundance of anaerobic and facultative anaerobic taxa during PEs.

**Fig. 4:**
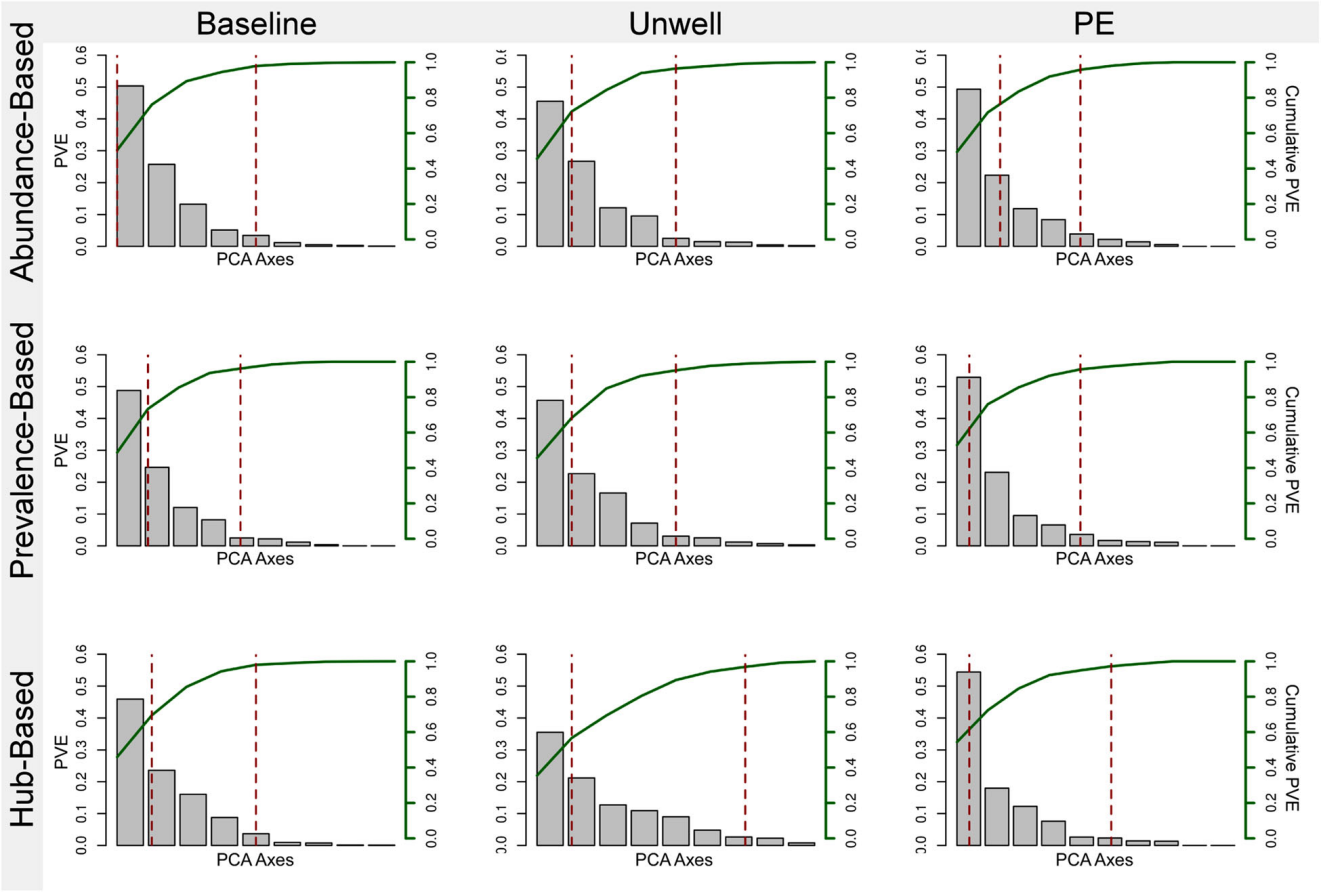
Principle coordinates analysis (PCoA) scree plots based on the diversity of key taxa identified by the three strategies (abundance-, prevalence-, and hub-based) for each clinical state (i.e. baseline, unwell, and pulmonary exacerbations). Each PCoA axis is plotted against the total percent variance explained (PVE) on the left y-axis, while the cumulative percent variance explained is plotted in green on the right y-axis. The two dashed lines in each plot correspond to the 50% and 90% cumulative PVE

We assessed the stability of each clinical state community as identified by the three strategies via principle coordinates analysis (PCoA). Our goal was to see how the clinical state was reflected in the variability of the community. Scree plots are shown in Fig. 4, with the PCoA axes plotted against the percent of variance explained (PVE; the two dashed red lines indicate 50% and 90% cumulative PVE, respectively). A dramatic difference can be seen in the pattern of variation in the PE state compared to the baseline and unwell states. In the PE state, a very small number of PCoA axes account for the majority of variance, indicating that a large amount of variation can be explained by a small number of variables (e.g. taxa). The difference is even more dramatic when considering the hub-based strategy, where 50% of the PVE can be explained by only 6% of the PCoA axes as opposed to over 36% of the axes for both the baseline and unwell states. The fact that the variation in the community is driven by a smaller number of taxa indicates that the PE community is more rigidly structured than the other two communities.

Finally, we measured diversity for the top ten key taxa identified in each of the three clinical states using the three selection strategies to assess associated changes between the community of key taxa and patient health (Fig. 2d). We found that diversity increased during PEs for both the abundance and prevalence-based strategies (yellow and green lines, respectively, in Fig. 2d), but, decreased when using the hub-based strategy (blue line in Fig. 2d). It is important to recognize that the number of taxa (i.e., taxa richness) examined in each case is held constant at ten, so a decrease in diversity indicates that the relative abundances of the key taxa in the PE community become more skewed, while an increase means that the relative abundances become more uniform.

## Discussion

The human microbiome shows significant variation in its specific composition, interconnectedness, and resiliency within and among individuals.^34–38^ We have focused on identifying which measure of variation in the CF lung microbiome most closely tracks changes in patient health. We then used this measure to identify key taxa associated with the specific clinical states. Key taxa are those that play a crucial role in the ecological structure and function of the community, regardless of their overall abundance or prevalence. We are particularly interested in identifying changes associated with pulmonary exacerbations (PEs), or the intermittent, acute worsening of patient health or lung function that requires antibiotic treatment intervention.^5^

To date, most studies have focused on the impact of classic CF pathogens, such as *Pseudomonas aeruginosa, Burkholderia cepacia* complex, and *Staphylococcus aureus*. These studies have implicitly or explicitly assumed that the most abundant or prevalent (which may be assumed to reflect persistence) taxa in a niche will also be the key taxa. We instead used an unsupervised approach on data collected longitudinally through changing clinical states to identify key taxa. Our change-point detection analysis determined that key taxa that identified based on their interconnectedness (i.e., hub taxa) tracked clinical changes better than key taxa identified based on abundance or prevalence. We also could identify taxa associated with distinct clinical states much more readily using network-based measures than abundance or prevalence. Finally, our analysis revealed that anaerobes and facultative anaerobes formed the preponderance of highly interconnected genera statistically associated with pulmonary exacerbation.

Anaerobic taxa have only recently been found in significant numbers in the CF lung,^39–47^ and still play a largely undefined role in CF disease development.^45^ In fact, different studies have ascribed diametrically opposed impacts for anaerobic taxa. Initially, the presence of anaerobes was associated with decreased lung inflammation, and higher, although more variable, lung function during PEs.^46,47^ Later work has focused on their possible detrimental role on CF disease development, including airway environmental remodeling promoted by the acidic byproducts of anaerobic fermentative metabolism, such as acetaldehyde and short chain fatty acids, and the pH-neutral 2,3-butanedione pathway that is triggered to avoid acid production.^18–20,48^ The growth of anaerobes and acidic remodeling of their local environment is believed to contribute to an uneven gas mixture throughout the lung (i.e. ventilation inhomogeneity), a dysregulated immune response, and increased airway inflammation and tissue damage.^21,46–49^

The contradictory conclusions regarding the clinical significance of anaerobes raise the question as to whether they play a positive, negative, or neutral role in the development of PEs. One simple explanation for these contradictory results is that early studies generally had relatively small sample sizes, and consequently, conclusions were often based on a fairly small number of observations. But a more likely explanation is that antibiotic treatment was a confounding variable in these studies. Many studies look at early treatment responses during the first few days of intravenous antibiotic therapy and do not consider the anaerobic activity and effects of broad spectrum antibiotics, often because antibiotic data is not available. Consequently, the result of anaerobes being associated with less inflammation and higher FEV1 during exacerbations may be confounded by differences in antibiotic selection according to the severity of PE (e.g., those with less severe PEs may be on regimens with less anti-anaerobic activity such as ciprofloxacin, rather than a broader spectrum antibiotic such as meropenem). Therefore, it would be informative if future studies could sample at time-points before IV antibiotics are instituted, as opposed to during treatment, when antibiotics have significant confounding effects.

A number of the anaerobic bacteria identified in this study have been previously isolated from the respiratory tract and studied for their role in disease. *Actinomyces, Selenomonas, Leptotrichia, Johnsonella, Prevotella* and *Propionibacterium* are anaerobic members of CF lung microbiome that are also found in oropharyngeal flora. Some of these oropharyngeal bacteria are capable of inducing positive regulation of *P. aeruginosa* virulence genes via a quorum sensing dependent mechanism.^50^ Moreover, microbial fermentation products produced by anaerobic bacteria can induce pyocyanin production that enhances virulence in *P. aeruginosa*.^24^ We also identified *Dialister*, which is an anaerobe known to cause infections in the respiratory tract.^51^ Finally, our validation data set identified *Gemella* as being associated with PEs, which is consistent with the original findings of Carmody et al.^6^ Unlike the PE state, the hub-based strategy found that the baseline and unwell states were associated with many aerobic bacteria. Interestingly, *Pseudomonas* was found to be not strictly associated with any of three clinical categories, which is consistent with previous findings.^7^

Recent work by Zaneveld et al.^38^ has addressed the impact of perturbations on microbiomes in general. They argue that many dynamic transitions follow stochastic rather than deterministic paths, and therefore result in shifts from unstable and highly variable community states. They coined the term the ‘Anna Karenina principle’ to describe their argument, based on the quote from Leo Tolstoy that “all happy families look alike; each unhappy family is unhappy in its own way.” A corollary of the Anna Karenina principle is that dysbiotic communities should show greater variation in microbiota composition than communities from healthy individuals. Interestingly, our data come to the opposite conclusion. We find that PE communities consistently show less variation than baseline communities. For example, our cross-sectional PCoA analysis using the top ten hub key taxa in the PE community found that 50% of variance was explained in only 6% of the axes, as opposed to 36% of axes for the baseline community. This relationship held regardless of the strategy used for selecting key taxa. The reason for this divergence from the Anna Karenina principle is not clear, although one intriguing possibility is that specific microbiological factors are responsible for PEs. A direct causal relationship may mean that CF lungs deterministically transition from one specific, stable community to an alternative stable community during the shift from baseline to PE. If this is true then it provides further stimulus to dissect the fine-scale microbiological dynamics that occur leading up to and during PEs.

While the community variance data may hint at a causal relationship, it is important to keep in mind that our results do not demonstrate a causal role for anaerobic taxa and PEs. Determining the causal basis for these changes, excluding potential confounders, and testing the hypothesized link between anoxia and PEs will require substantial work, but may ultimately provide microbiological targets that enable the early detection and treatment of PEs. Nevertheless, the observation that the microbial community is restructured during the transition from the baseline clinical state to PE, and that anaerobes are associated with PEs provides leads for identifying biomarkers associated with the clinical state of CF patients, similar to what has been proposed by others.^52–54^

In conclusion, this study provides a means to identify microbes of interest in dynamic environments by assessing their interaction with other members of their community and correlations between changes in their abundance with changes in an environmental variable of interest. In this analysis, we found a strong correlation between anaerobic microbes and PEs. This correlation lends further support to prior studies that have postulated that anaerobic microbes and the development of anoxic conditions in the CF lung are associated with PEs. Further, the observation that anaerobes are associated with PEs suggest that selectively targeting anaerobic microbes and fermentative metabolism may reduce the occurrence or severity of PEs. Potential ways of doing so may include the use of antibiotics such as metronidazole that more specifically target anaerobes, or the use of hyperbaric oxygen to disrupt the anaerobic feedback cycle that leads to fermentative metabolism and increased lung damage.

## Methods

### Study information, data sets and microbiome analysis

Two hundred and sixty-six expectorated sputum samples were collected from 18 CF patients (range 8–60 samples / patient, mean = 14.1 ± 11.92 s.d., median = 11) from St. Michael’s Hospital and the Hospital for Sick Children in Toronto, Canada. This study received approval from the research ethics boards of both hospitals. Patients provided informed written consent and were enrolled prospectively at The Hospital for Sick Children and St. Michael’s Hospital between 2010 and 2013. Expectorated sputa were collected for all patients. The clinical state of patients in the discovery data set was determined in the clinic at the time of visit and samples were binned into one of three clinical states based on the physician’s diagnosis at the time of the patient's clinic visit: (a) baseline, (b) unwell, and (c) PE. Detailed patient data can be found in Coburn et al.^7^ Pulmonary exacerbation was defined as the acute worsening of patient health that required intravenous antibiotic therapy. Baseline state was defined when the patient was stable or fully recovered and did not need treatment beyond maintenance therapy. The unwell clinical state was assigned to cases where the patient was not diagnosed with a PE (i.e., there was not acute worsening of patient’s health), yet the patient was judged to be ill by the physician. While milder cases of unwell were left untreated, in some cases patients were put under antibiotic therapy to speed their recovery as well as prevent the development of a PE.

We also used an alternative clinical classification scheme proposed by Zhao et al.^10^ known as the BETR system to ensure that our results were robust to the specific clinical classification scheme employed. This system classified patients into the following: (1) Baseline - no antibiotics for at least 30 days; (2) Pulmonary Exacerbation - physician defined requiring antibiotic treatment; (3) Treatment - intravenous or oral antibiotic treatment; and (4) Recovery - off antibiotics for less than or equal to 30 days.

For the discovery data set, sequencing was performed at the University of Toronto, Centre for the Analysis of Genome Evolution and Function (CAGEF). Microbiome analysis was performed by analysis of the V5 to V7 hypervariable regions of the 16S rRNA gene using the SI-Seq protocol.^28^ USEARCH^55^ was used to detect and remove the chimeric reads as well as pick OTUs against a SI-Seq structured reference set using a similarity of 0.87. Taxonomic assignment was performed using an RDP structured data set using UCLUST with –max_accepts set to 0 and similarity set to 0.97 (empirically determined to produce the highest consistency identification at the genus level using *P. aeruginosa* controls). For more detail on the experimental protocols see Maughan et al.^28^ OTU relative abundance and patient clinical status data are available in Supplementary Table 1.

The validation data set was produced by Carmody et al.^11^ Raw 16S rRNA read sequences were downloaded from NCBI SRA (SRA: SRP051730; BioProject: PRNJA271691) and standard QIIME^56^ protocol was used for microbiome analysis and taxonomy assignment.

### Network construction

To construct the microbiome co-occurrence network from OTU tables, we first calculated a measure of relationship between each pair of OTUs in the microbiome, followed by a decision on which interaction qualifies to be included in the microbiome network, based on a threshold or a measure of confidence. Here, we used the partial correlation to measure the level of association between pairs of OTUs. Partial correlation measures the degree of association between two random variables excluding the effect of a set of controlling random variables. Unlike Pearson correlation, partial correlation of two OTUs calculates the amount of correlation left after eliminating the influence of other OTUs. We then estimated the local false discovery rates and p-values for the correlation coefficients, as our null model. Next, we removed the edges with p-values higher than our decision threshold, which was 0.05 for this study. The microbiome network construction was carried out in R using “FDRnetwork” function of “qgraph”^29^ package. However, It should be noted that there are a multitude of methods available to construct co-occurrence networks from OTU tables, which employ a variety of statistical measures to compute the associations between taxa, ranging from simple Pearson correlation coefficient to advanced probabilistic graphical models methods (for a detailed review of network construction and analysis methods applicable to microbiome, see ref. ^57^).

### Hub detection

We applied the PageRank algorithm^30^ to the microbiome networks to identify key members of CF lung microbiome in each patient. PageRank is a link analysis algorithm with the underlying assumption that hubs are likely to be more connected to other nodes when compared to non-hub nodes. This algorithm assigns a numerical weight to each node of a network as a measure of its relative importance within the network. The numerical weights of the nodes (also called PageRanks of the nodes) were then sorted and the top five or ten taxa with highest PageRank were selected as microbiome network hubs (or key taxa). This whole process was performed in R using “igraph” package.^58^

### Change-point detection

We used the pruned exact linear time (PELT) algorithm^31^ to search for change-points in the abundance of key microbiome members. The PELT algorithm is exact (i.e., always finds the optimal solution for the problem) and has a computational cost that is linear to the number of data points. This dynamic programming approach tries to divide data into segments and fit a parametric model to data within each segment, so that one segment does not depend on the parameters in another segment. This will define the number and position of the change-points. To avoid overfitting, the optimal segmentation is found by minimizing the cost of segmentation. PELT adds data points one at a time to calculate the optimal segmentation and involves a pruning step that reduces the computational cost of the method without affecting the exactness of the resulting segmentation. Moreover, it is shown to lead to a substantially more accurate segmentation than other change-point detection algorithms and can be applied to find change-points under a range of statistical criteria such as penalized likelihood, quasi-likelihood and cumulative sum of squares. Here the change-point analysis was performed in R using “PELT” function of “change-point” package with default parameters.^31^

### Association measures

In order to measure the level of association between microbiome composition and patients’ clinical states, we calculated the cross-covariance between the change-points found in the abundance of key microbiome members and the changes in the clinical state of the patient. Given two stochastic processes, the cross-covariance is a function that gives the covariance of one process with the other at pairs of time points. Given the nature of our change-point and clinical states data (i.e., representing changes in abundance of key microbiome members and clinical state of patients, respectively, at various time points), cross-covariance was chosen as the most appropriate statistical metric to measure the association between these two types of data. Here, the cross-covariance measures were calculated in R using “ccf” function of the “stats” package. We also employed the non-parametric Mann-Whitney-Wilcoxon test to decide whether the distributions of cross-covariance measures obtained by the three strategies were identical (i.e. null hypothesis), without assuming them to follow the normal distribution. To test the hypothesis, we applied the “wilcox.test” function of R’s “stats” package to the cross-covariance measures obtained for different strategies. We then rejected the null hypothesis at p-values less than the 0.05 significance level (e.g. asterisks in Fig. 1c, d).

### Diversity index measurements

To quantify the amount of variation captured by each key taxa identification approach, we used two widely used diversity indices: Shannon and Inverse Simpson.^59,60^ Shannon's diversity index quantifies the entropy, which represents the uncertainty about the identity and grouping of an unknown entity. The Inverse Simpson index measures the degree of concentration when entities are classified into groups by calculating the probability that two entities taken at random from a data set of interest represent the two different groups. We used the “diversity” function in R’s “vegan” for the calculations.

### Regression analysis

To compare our data sets, we applied a regression analysis to the cross-covariance measures (between changes in relative abundance of key taxa and the changes in the clinical state of patients) obtained for key taxa in the two data sets. For each approach, we constructed a linear regression model and applied it to the cross-covariance measures computed for both discovery and validation data sets. Furthermore, we combined the measurement from all the three strategies and applied a linear regression model to them to see the overall relationship between the two data sets. For each model, R-squared coefficient of determination and root-mean-square error (RMSE) was calculated. RMSE is the square root of the mean of the square of all of the error that measures how spread out the residuals (i.e. prediction errors) are around the line of best fit. It makes an excellent general-purpose error metric for numerical predictions and compared to the similar mean absolute error, RMSE amplifies and severely punishes large errors. Moreover, we used a support vector regression (SVR) model to calculate the minimum RMSE error for each model. SVR is a nonparametric technique that relies on nonlinear kernel functions (i.e. the functions that transform the data into a higher dimensional feature space to make it possible to perform the linear separation). Linear and SVR models were built in R using “lm” function of “stats” package and “svm” function of “e1071” package, respectively.

### Principal coordinate analysis (PCoA)

Principal Coordinates Analysis (PCoA, = Multidimensional scaling, MDS) was used to explore and visualize dissimilarities or differences between individuals and groups. We used R’s “vegan” package to calculate pairwise Bray-Cutis distances within our log-transformed data. Then, “pcoa” function from R’s “ape” package was used to obtain eigenvalues and eigenvectors. To correct for negative eigenvalues generated during decomposition (a known by-product of non-Euclidean distance metrics), we used Lingoes procedure. This correction procedure adds a constant value, equal to twice the value of the largest negative value, to any original squared distance that is not diagonal.

### Code Availability

We have provided R code and example data files to perform the analysis discussed in this study. Two scripts are provided: netcp.R performs network analysis and change-point detection; utils.R contains accessory functions (see Supplementary Software).

## Supplementary information

Supplemental Figures

R code

R code

Supplemental Data Tables

Supplemental Data Tables

## Data Availability

The data used for the discovery and validation sets are available through NCBI SRA accession numbers SRP135694 and SRP051730, respectively.

## Supplementary information

**Supplementary information** accompanies the paper on the *npj Biofilms and Microbiomes* website (10.1038/s41522-018-0077-y).
