# Supplementary material for: Microbiome networks and change-point analysis reveal key community changes associated with cystic fibrosis pulmonary exacerbations
Source: NPJ Biofilms Microbiomes. 2019 Jan 21;5:4. doi: 10.1038/s41522-018-0077-y (PMC6341074; doi:10.1038/s41522-018-0077-y)
Supplement: Supplementary file 1 — Supplemental Figures [file 41522_2018_77_MOESM1_ESM.pdf]

SUPPLEMENTARY INFORMATION

Microbiome Networks and Change-Point Analysis Reveal Key Community Changes Associated with Cystic Fibrosis Pulmonary Exacerbations

Mehdi Layeghifard, Hannah Li, Pauline W. Wang, Sylva L. Donaldson, Bryan Coburn, Shawn T. Clark, Julio Diaz Caballero, Yu Zhang, D. Elizabeth Tullis, Yvonne C. W. Yau, Valerie Waters, David M. Hwang, David S. Guttman

Supplementary Figures

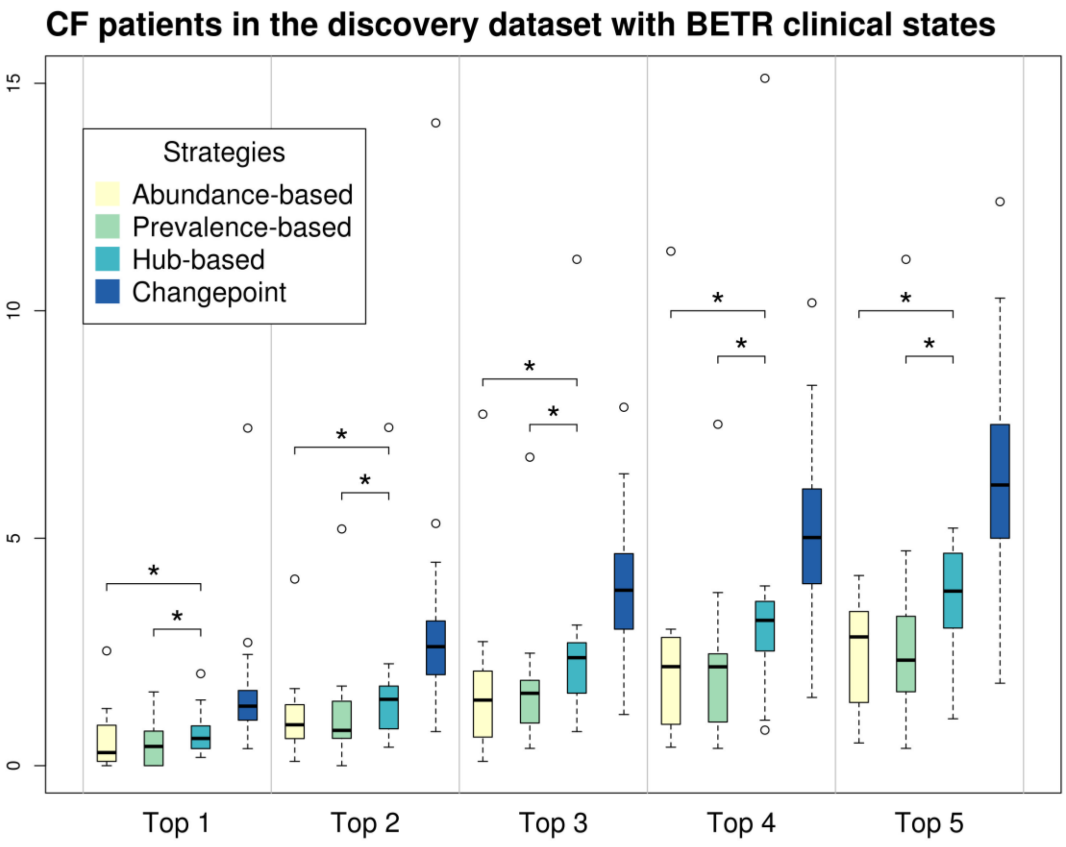

Supplementary Figure 1. Cumulative cross-covariance measures of association between key taxa and clinical states of CF patients (the discovery dataset) using the alternative BETR system. key taxa were identified using three strategies: an abundance-based approach that identifies key taxa as those with the highest overall relative abundance (AB); a prevalence-based approach that identifies key taxa as those with the highest prevalence across all samples; and a hub-based approach that identifies key taxa as those that are most central to the microbiome networks (i.e., highest degree of interconnectedness, HB). We also identified the five taxa whose changes in relative abundance showed the strongest association to changes in clinical state for each patient. This change-point standard represents the strongest association possible in the dataset, and is therefore useful as a metric, but is of no predictive value since it requires prior knowledge of the clinical state. The association measures were estimated between change-points in the relative abundance of key taxa, as determined by the four strategies and change-points in the clinical state of the patients. The y-axis represents the cumulative sum of cross-covariances for five categories shown on the x-axis, representing key taxa. These categories represent top one to top five ranked key taxa found by each of the four strategies. The 'Top 1' category shows the cross-covariance measures of association between the changes in relative abundance of the most important taxon identified by each of the four strategies and changes in the clinical states of CF patients. The 'Top 2' through 'Top 5' categories show sums of similar cross-covariance measures of association for the top two through top five highest ranked taxa. The Mann-Whitney-Wilcoxon test was used for statistical hypothesis testing ($p < 0.05$; The asterisks represent the significant differences between HB and AB and PB strategies).

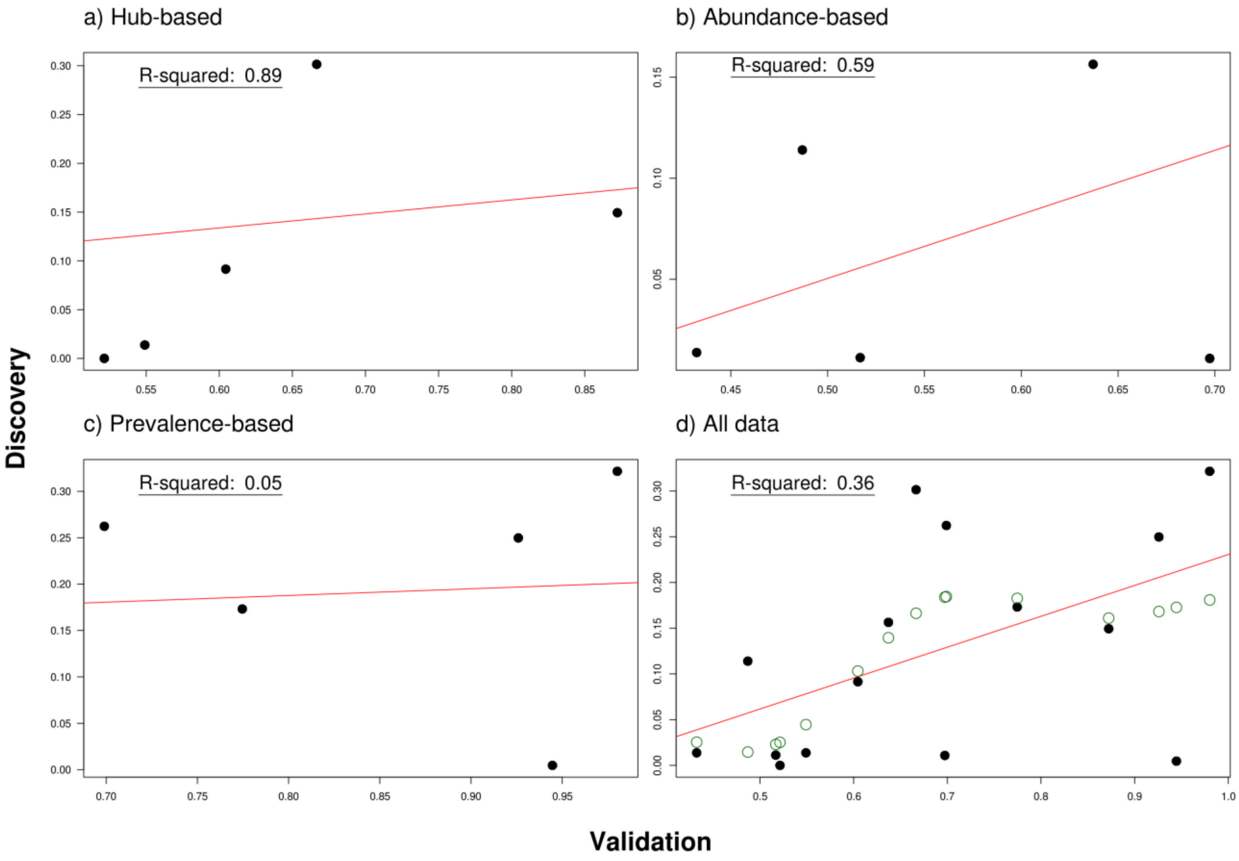

Supplementary Figure 2. Regression analysis between discovery and validation datasets using top five key taxa. Cross-covariance measures of associations between changes in relative abundance of top five key taxa and patients' clinical states were used in this analysis. a-d) Regression analysis between the two datasets using top five key taxa for the three strategies used to identify key taxa (a-c) and for the results of all the strategies combined (d). R-squared indicates the coefficient of determination. For comparison, the best possible fit found by SVR is represented by a set of green hollow circles in (d).

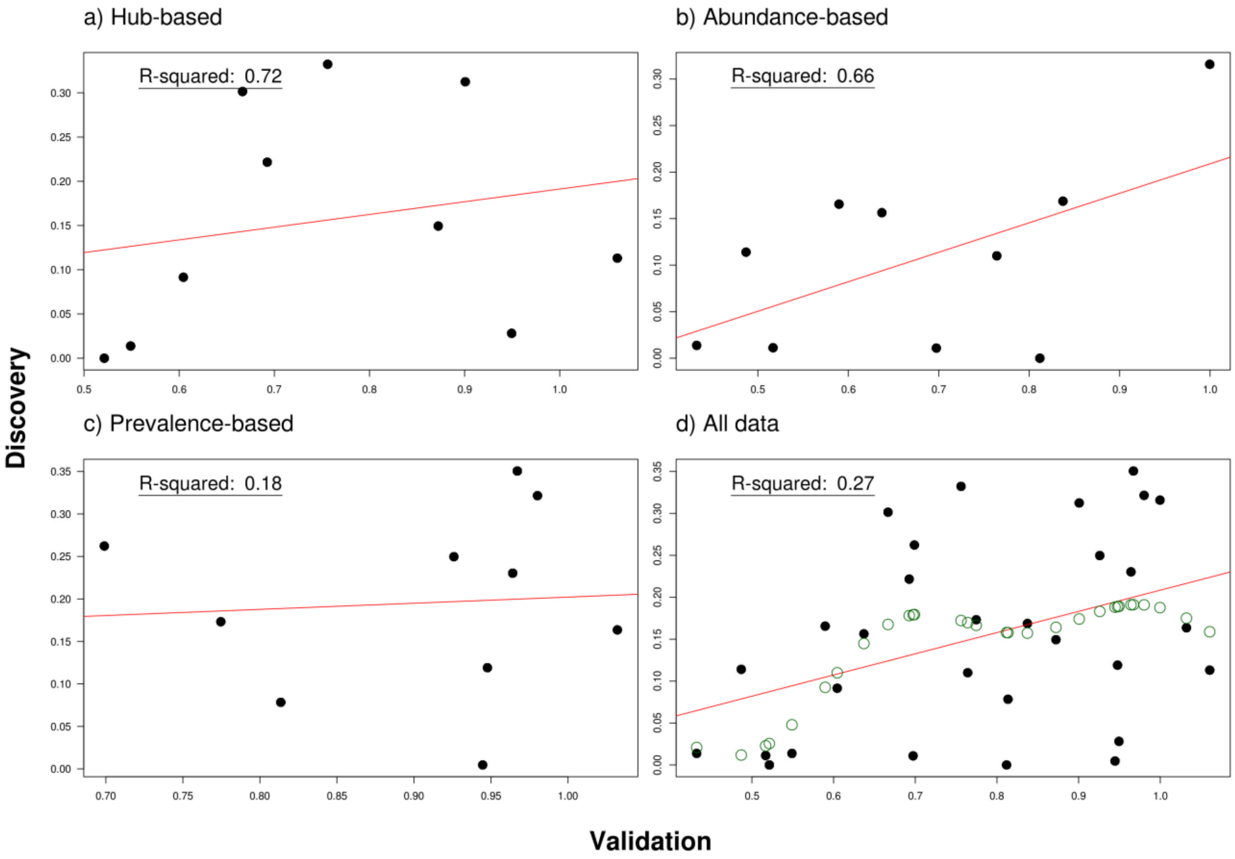

Supplementary Figure 3. Regression analysis between discovery and validation datasets using top 10 key taxa. Cross-covariance measures of associations between changes in relative abundance of top 10 key taxa and patients' clinical states were used in this analysis. a-d) Regression analysis between the two datasets using top five key taxa for the three strategies used to identify key taxa (a-c) and for the results of all the strategies combined (d). R-squared indicates the coefficient of determination. For comparison, the best possible fit found by SVR is represented by a set of green hollow circles in (d).

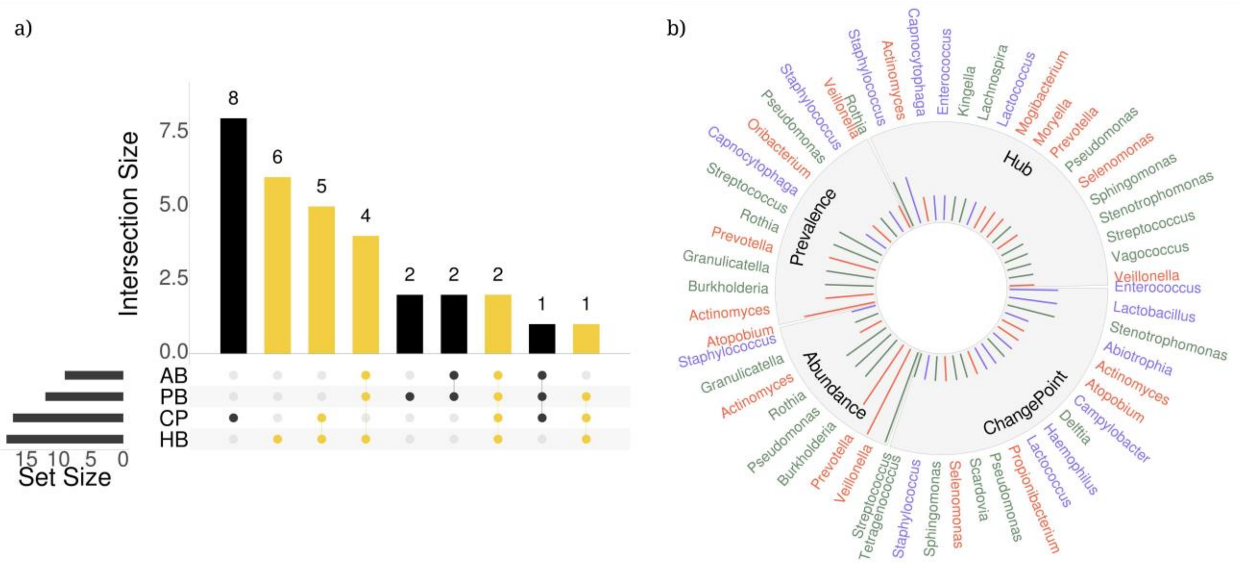

Supplementary Figure 4. The relationships between the different key taxa selection strategies and the taxa identified by each within the validation dataset. a) An UpSetR visualization of interactions between sets of key taxa identified by the four different key taxa selection strategies (abundance-based, AB; prevalence-based, PB; hub-based, HB, and the change-point standard, CP). The grid along the bottom is used to identify interaction sets (analogous to a Venn diagram). A heavily colored yellow or black dot in the grid indicates that the corresponding key taxa selection strategy is included in that set. Thin vertical lines connecting dots indicate that multiple strategies are included in the set. The black horizontal bars on the left indicate the number of taxa identified by each selection strategy. The large vertical bars on top indicate the number of taxa in each set. By way of example, the first bar on the left indicates that 15 key taxa were identified only by HB strategy, while the second bar indicates that 11 taxa were identified by both HB and CP strategies. b) All the key taxa identified in the validation dataset stratified by the key taxa selection strategy. Taxa are color-coded based on their mode of metabolism (green, blue, and red for aerobic, facultative, and anaerobic microbes, respectively). The bar radiation from the center of the chart indicates the relative frequency of each key taxon among all the patients in the dataset.

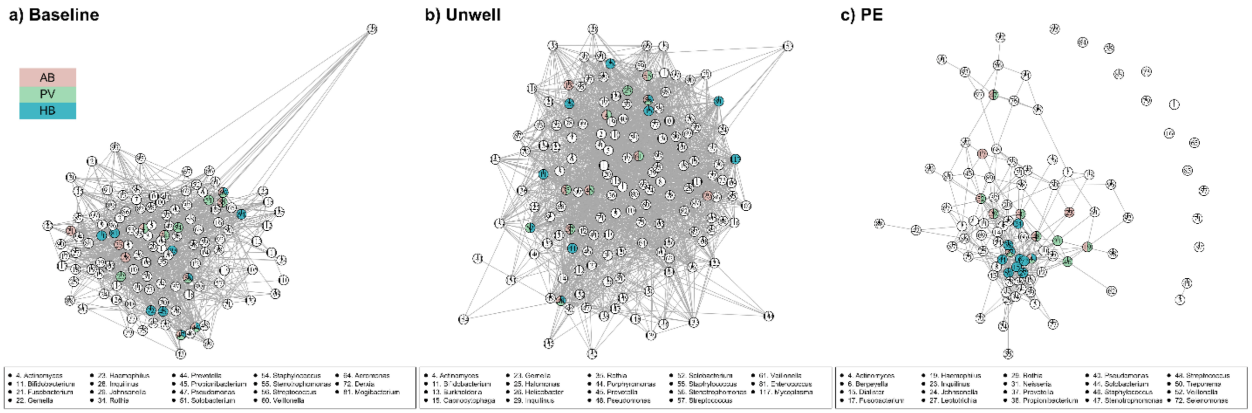

Supplementary Figure 5. Co-occurrence networks of CF lung microbiome stratified based on the patients' clinical states in the cross-sectional analysis. Taxa at the genus level are represented by hubs, while co-occurrence is represented by edges connecting hubs. a-c) Networks reconstructed using relative abundance of taxa present in the baseline, unwell and PE clinical states, respectively, across all patients. The top 10 key taxa detected by abundance-based (AB), prevalence-based (PB), and hub-based (HB) strategies are identified in the legends below the networks by name and the corresponding node number in the networks. These key taxa are also highlighted in the networks using colored pie charts (see color legend at top left), while non-key taxa are identified by white circles.

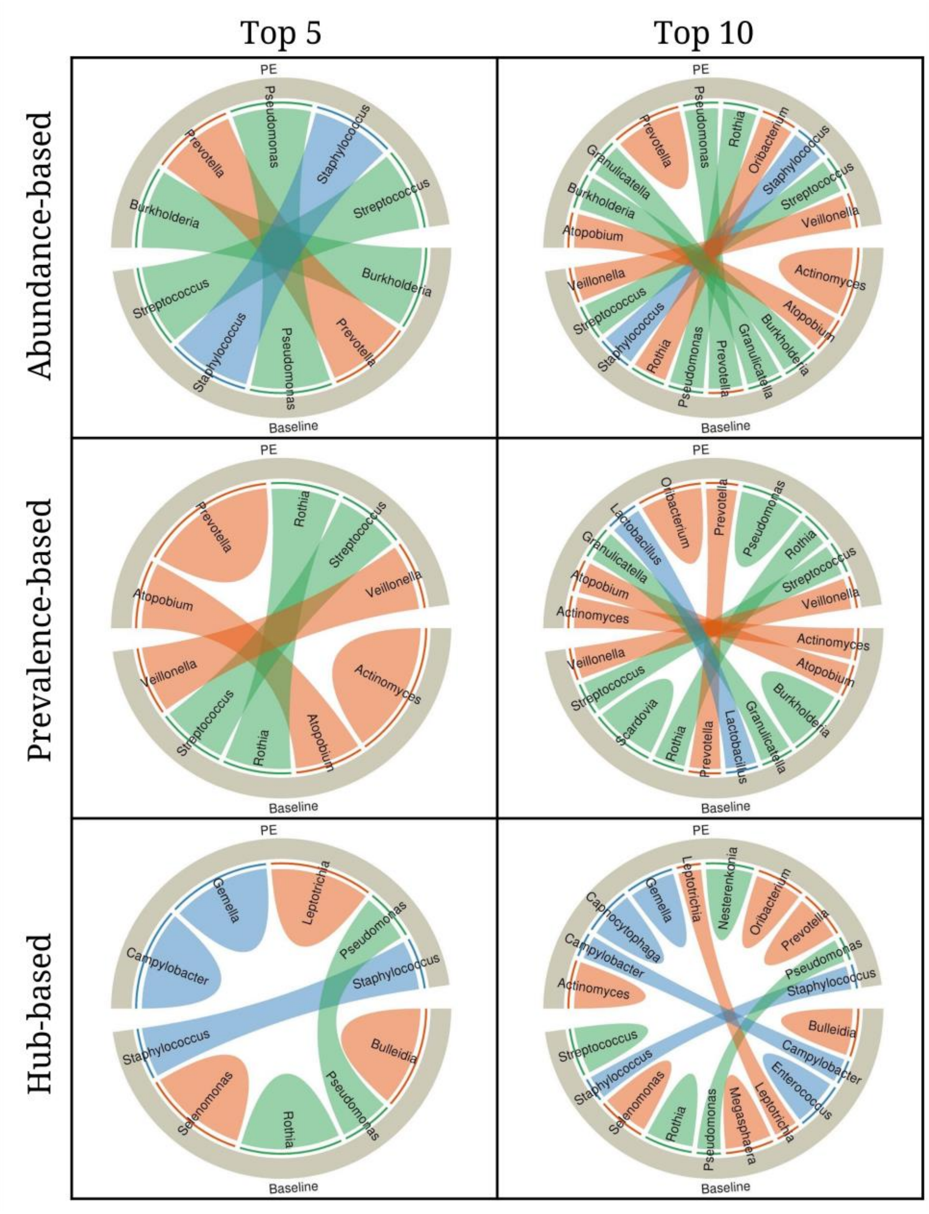

Supplementary Figure 6. Cross-sectional analysis of the validation dataset. Associations between key taxa when samples are stratified into two clinical states using a cross-sectional analysis for both the top five and top ten key taxa in the validation dataset. The chord diagrams show the key taxa identified by the three different strategies (e.g. abundance-based, prevalence-based, or hub-based). The outer ribbon identifies the respective clinical states, and encompasses the set of key taxa identified to be associated with each state. Taxa that are associated with more than one state are connected by chords in the inner circle. Taxa and their respective chords are colored based on their primary mode of metabolism, with red, blue, and green representing anaerobic, facultative anaerobic, and aerobic taxa, respectively. Fewer interactions (i.e. chords) between clinical states demonstrate the higher level of stratification of key taxa based on patients' health, i.e. the power of each strategy to delineate key taxa based on patients' clinical states.

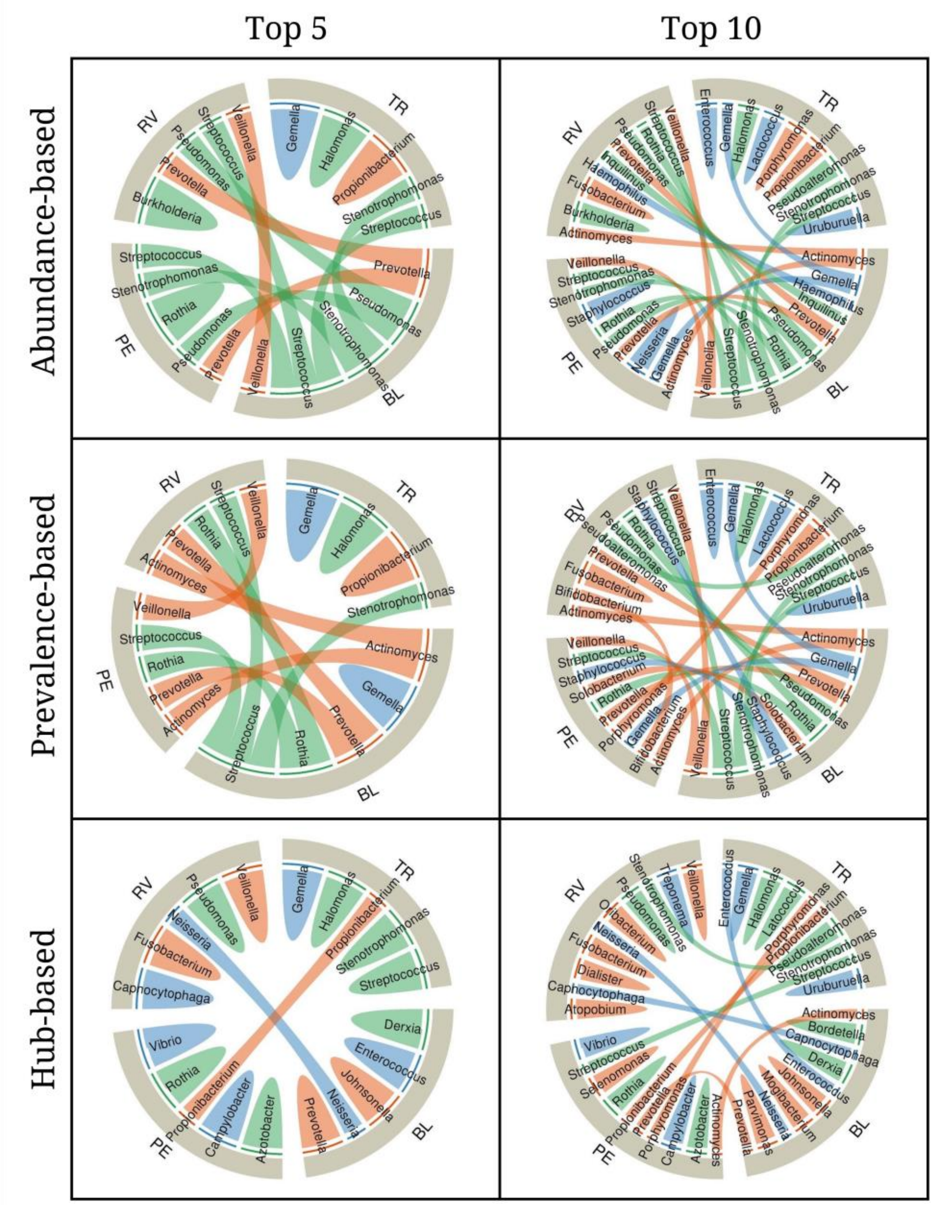

Supplementary Figure 7. Cross-sectional analysis of the discovery dataset using the BETR clinical classifications. Associations between key taxa and BETR clinical states (baseline, PE, treatment, recovery) in a cross-sectional analysis for both the top five and top ten key taxa in the discovery dataset. The chord diagrams show the key taxa identified by the three different strategies (e.g. abundance-based, prevalence-based, or hub-based). The outer ribbon identifies the respective clinical states, and encompasses the set of key taxa identified to be associated with each state. Taxa that are associated with more than one state are connected by chords in the inner circle. Taxa and their respective chords are colored based on their primary mode of metabolism, with red, blue, and green representing anaerobic, facultative anaerobic, and aerobic taxa, respectively. Fewer interactions (i.e. chords) between clinical states demonstrate the higher level of stratification of key taxa based on patients' health, i.e. the power of each strategy to delineate key taxa based on patients' clinical states.
